# Ethnic Socialization, Ethnic Identity, and Self-Esteem in Chinese Mulao Adolescents

**DOI:** 10.3389/fpsyg.2021.730478

**Published:** 2021-10-22

**Authors:** Lu Kuang, Saori Nishikawa

**Affiliations:** ^1^Psychological Health Center, Guilin University of Electronic and Technology, Guilin, China; ^2^Graduate School of Social and Cultural Sciences, Kumamoto University, Kumamoto, Japan

**Keywords:** ethnic socialization, ethnic identity, self-esteem, Mulao, adolescents

## Abstract

We examined the associations and likely pathways between ethnic socialization, ethnic identity, and self-esteem among junior high school students of Chinese Mulao ethnic minority. A total of 469 Mulao students (220 boys and 249 girls) completed the Ethnic Socialization Measurement revised by Yin et al. (2016), the Revised Multigroup Ethnic Identity Measure (MEIM-R) by Roberts et al. (1999), and Rosenberg’s Self-esteem Scale (Chinese Version) by Wang et al. (1999). The main results indicated that adolescents who perceived more promotion of harmony messages tended to report stronger ethnic identity and higher self-esteem. Adolescents who perceived cultural socialization displayed stronger ethnic identity and higher self-esteem, while the promotion of distrust messages was negatively associated with self-esteem. Multiple-group analysis revealed that the relationships were stable across gender, parental education, but varied significantly across students’ grade. These findings emphasize the important role of positive ethnic socialization messages in adolescents’ ethnic identity and self-esteem. In addition, it is also important that we pay attention to negative ethnic socialization messages and consider their grade when communicating ethnic information with adolescents. Finally, our results are analyzed and notable suggestions are presented for ethnic family education.

## Introduction

### Ethnic Socialization

Considering the increasing researches of ethnic socialization, and the close communication among different ethnic minority groups, our current study priority was to clarify how ethnic socialization affect ethnic minority adolescents’ identity and their self-esteem. Researches on ethnic-racial socialization nearly emerged over 47 years ago and predominantly focused on American ethnic minorities (Wilkinson, 1974; Bowman and Howard, 1985; Peters, 1985; Rodriguez et al., 2009). More recently, this field has expanded to other minority groups, such as American adopted children (Mohanty and Newhill, 2011; Mohanty, 2013; Rosnati and Ferrari, 2014; Hu et al., 2017), as well as European Roma ethnic minority youth (Dimitrova et al., 2018). According to Hughes and Johnson (2001), ethnic socialization is considered as how parents shape children’s learning about their own race and their relationships with other ethnic groups. Parents are the crucial agent of ethnic socialization, besides peers (Rivas-Drake et al., 2009), neighborhood and community (Smith et al., 2003) are also play important role. Communication with children about their ethnicity is the main and salient part of ethnic family education. Conceptually, ethnic socialization messages have often been categorized into three categories: cultural socialization, preparation for bias, and promotion of distrust (Hughes et al., 2006; Juang et al., 2017). The latter two categories are aimed at raising awareness and preparation for negative things that minority ethnic adolescents may encounter in the future (Hughes and Chen, 1997), while the first category message is to promote positive messages concerning the ethnic group’s culture (Boykin and Toms, 1985; Hughes et al., 2006; Atkin and Yoo, 2019; Ulerio and Mena, 2020).

### Ethnic Socialization in Adolescents

The past studies mainly conducted among the adolescents who aged from 12 to 18 years old (Hughes and Johnson, 2001; Hughes, 2003; McHale et al., 2006). According to Erikson (1968), ego identity is the most important topic during this period, adolescents continually explore their social role and seek the meaning of themselves in this stage of the life cycle. Ego identity is established after exploring multiple options and making a commitment to the choice made (Marcia, 1966, 1967).

Recent studies of ethnic socialization have mainly focused on *cultural socialization* messages, and have revealed that such messages are positively correlated with minority adolescents’s performance (Bynum et al., 2007; Seol et al., 2016), such as self-esteem (Lee et al., 2018) and ethnic identity (Juang and Syed, 2010). According to Gartner et al. (2014), cultural socialization messages are correlated with higher ethnic identity and subsequently promote self-esteem among Asian-American adolescents. Regarding the association between *preparation for bias* messages and ethnic identity, there is a lack of consensus in the empirical results. According to Rivas-Drake (2011), Latin college students who perceived preparation for bias messages showed negative public regard, which was the main dimension of ethnic identity. Gartner et al. (2014) found that the association between preparation for bias and ethnic identity was not significant among Asian-American adolescents. These correlations have not been sufficiently verified in existing studies. Perceived *promotion of distrust* messages in the process of ethnic socialization is aimed at raising children’s vigilance when they get along with people from other ethnic backgrounds. The promotion of distrust is negatively correlated with ethnic identity (Gartner et al., 2014). In addition, Yin et al. (2016) investigated 10,390 Chinese minority students and found that their ethnic socialization messages showed unique cultural features that could be mainly divided into four categories: cultural socialization, promotion of distrust, preparation for bias, and promotion of harmony. Notably, *the promotion of harmony* dimension was aimed at promoting a positive relationship with other groups, and it was positively correlated with ethnic identity (Yin et al., 2016).

### Ethnic Identity

Ethnic identity is defined as self-identification as a group member, a sense of belonging, and an attitude toward the group (Phinney, 1992, 2003). In addition, self-identity is an important component of self-concept, especially among adolescents (Erikson, 1968). Furthermore, ethnic identity is one aspect of social identity for minority adolescents (Tajfel, 1981) and also a common phenomenon across groups (Phinney, 1992). Over the past three decades, ethnic identity has been the most commonly researched outcome of parents’ ethnic socialization (Brittian et al., 2013). Based on the Theory of identity development (Phinney and Ong, 2007), ethnic identity is developed from the process of commitment and exploration. Commitment mainly refers to the strong attachment and personal investment toward a group. Exploration means the search for ethnic information and activities, such as reading and talking about things related to the group, understanding ethnic culture, participating in cultural activities. These process correspond with the information of ethnic socialization, especially for the culture socialization. Hence, it is reasonable to speculate that ethnic socialization may have a effect on ethnic identity. Additionally, previous research also widely provide empirical support for the correlations between ethnic socialization and identity (Brega and Coleman, 1999; Umaña-Taylor and Fine, 2004; Brittian et al., 2013; Gartner et al., 2014; Reynolds and Gonzales-Backen, 2017; Dimitrova et al., 2018). Besides, according to Rodriguez et al.’s (2009) model, ethnic identity is also a predictor of minority adolescents’ psychological and educational outcomes.

### Self-Esteem

Self-esteem, a one-dimensional construct, is defined as a general sense of one’s worth (Rosenberg, 1965). It refers to how people value themselves, especially their feelings about their own social status, and how much coordination exists between their ideal and real selves (O’Donnell et al., 2008; Cieślak and Golusiñski, 2018). Ethnic identity, which is a sense of belonging and identification to a group. Social identity theory (Tajfel and Turner, 1986) pointed out that individuals always have an potential desire for social identity which is developed from the processes of comparison, categorization, and identification. Ethnic minorities will compare the inner-group with the other groups and then conclude the difference and similarities. Categorization will make them feel that they are similar to others. Hereby, a sense of identity will be generated, which results in the preference of the inner group. Self-esteem is affected to a certain extent by individual group identity or social identity which means belonging and pride. Existing studies also noted that ethnic identity could be a predictor of self-esteem (Smith et al., 1999; Umaña-Taylor and Fine, 2004; Umaña-Taylor and Updegraff, 2007; Mohanty, 2013; Gonzales-Backen et al., 2015).

### Moderating Role of Age, Gender, and Parents’ Education

Overall, Previous studies have reported that age, gender, and parents’ education could be predictors of ethnic socialization (Bowman and Howard, 1985; González et al., 2006; Hughes et al., 2006; Priest et al., 2014). Parents may tend to engage in some certain type of messages according to child’s age (Hughes and Chen, 1997), more variation in age is necessary to observe an effect (Brown et al., 2007). Meanwhile, where findings of ethnic socialization varied, gender was a considerable factor (Bowman and Howard, 1985; Caughy et al., 2002; Brown et al., 2007; Chavous et al., 2008; Cunningham et al., 2012). Additionally, parents is the primary ethnic socialization agent, whose education level affected their parental style (White-Johnson et al., 2010). These variables may moderate the relationships between ethnic socialization, ethnic identity, and self-esteem. Further, Brown et al. (2009) examined the role of gender and provided supporting evidence for the moderating effect on the association between ethnic socialization and academic grades. Similarly, a significant gender difference in path from cultural socialization to identity was showed in the research from Gartner et al. (2014).

### Limitations of Previous Studies

Previous studies mainly adopted the whole perspective when considering the influence of ethnic socialization and viewed it as one latent variable (Supple et al., 2006; Lee et al., 2018; McDermott et al., 2018). However, considering multidimensional ethnic socialization, different ethnic messages may have different correlations with ethnic identity. Studies have proposed the three categories of messages should be considered as three observed variables rather than one whole latent variable as “ethnic socialization” (Liu and Lau, 2013; Gartner et al., 2014). Gartner et al. (2014) proposed a method that considers the influence of ethnic socialization deeply and separately. Most studies on ethnic socialization have predominantly been conducted in the United States (94%) (Umaña-Taylor and Updegraff, 2007; Priest et al., 2014; Ulerio and Mena, 2020). Therefore, it is necessary to examine ethnic socialization theory in the context of Eastern countries (Atkin and Yoo, 2019). Additional studies on ethnic socialization from diverse groups would contribute to the understanding of the process (Hughes et al., 2006). The Mulao minority group is a traditional ethnic group in China that retains their unique culture and customs (Wu, 2016). Focusing on this group’s ethnic socialization process could provide more empirical evidence to the associations between ethnic socialization and adolescents’ mental performance from different cultural backgrounds.

### Current Study

The first aim of this study was to examine the relationships between ethnic socialization, ethnic identity, and self-esteem among Chinese Mulao minority adolescents, which could reveal the influence of ethnic socialization.

Second, we examined the links between ethnic socialization and self-esteem via ethnic identity to ascertain its stability in terms of different genders, grades, and parents’ education. Based on the theoretical and empirical evidence, we hypothesized the following: (1) Positive ethnic socialization messages (cultural socialization, promotion of harmony) would be positively correlated with ethnic identity. (2) Negative ethnic socialization messages (preparation for bias and promotion of distrust) would be negatively associated with ethnic identity. (3) Ethnic identity would be positively correlated with self-esteem and mediate the relationships between ethnic socialization and self-esteem. (4) Gender, grade, and parental education would moderate the relationship between perceived ethnic socialization, ethnic identity, and self-esteem.

## Materials and Methods

### Participants

The participants were Mulao ethnic minority adolescents residing in the Luocheng Mulao Autonomous County. The original sample consisted of 530 students. A total of 61 students were eliminated due to more than 50% of total items not being answered, or more than 90% of answers were the same, or answered regularly. Finally, 469 valid samples remained, with a response rate of 88%.

The mean age of the sample students was 14.43 years (range = 11–17 years; *SD* = 1.07), consisting of 220 (44.4%) boys and 249 (55.6%) girls. In addition, 131 (28.0%) students were freshman students, 132 (28.1%) were junior students, and 206 (43.9%) were senior students. The description of sample characteristics is presented in Table 1.

**TABLE 1:** The description of the original sample characteristics based on gender, grade, parental education level.

**Variables**	**Category**	***n***	**(%)**
Gender	Boys	220	46.9
	Girls	249	53.1
Grade	First grade	131	28.0
	Junior grade	132	28.1
	Senior grade	206	43.9
Parental education level	Primary school	159	33.9
	Junior high school	249	53.1
	Senior high school and above	61	13

### Procedures

Participants were recruited from three junior high schools in different townships of Luocheng Mulao Autonomous County, which is their major residential area. The school authorities were contacted to obtain necessary consent for this study and permission was also secured from our affiliated university’s institutional review board. Participants were given 20 minutes to complete the survey during class time under the guidance of research team members, and questionnaire was collected on the spot. Only private information about age, sex, grade, and parents’ education was marked, and students were free to answer or withhold consent.

### Measures

#### Ethnic Socialization

The Ethnic Socialization Scale (ESS) was developed by Hughes and Johnson (2001) and is widely used in Europe and North America. The original ESS consisted of three subscales: cultural socialization (e.g., Talked with you about important historical events of your ethnic group), the preparation for bias (e.g., Told you must be better to obtain the same rewards because you are an ethnic minority), promotion of mistrust (e.g., Told to keep away from other groups because they may have discrimination against you).

Yin et al. (2016) revised this scale based on the Chinese cultural background and added the “promotion of harmony” sub-scale. This subscale contains information such as “communicated with you about the friendship between your group and other ethnic minorities.” The Revised Ethnic Socialization Scale has 16 items and shows good psychometric properties. In this questionnaire, students were asked to think about their communication with their parents in the past year and report the frequency of their parents talking about the specific issues. The participants responded on a five-point Likert scale ranging from 1 = *never* to 5 = *always*. Higher scores reflected more perceived ethnic socialization messages. The internal consistency of the revised ESS was 0.86, and the reliability coefficient of the four sub-scales ranged from 0.72 to 0.85.

#### Ethnic Identity

Phinney (1992) developed the Multigroup Ethnic Identity Measure (MEIM) based on identity development theory (Erikson, 1968) and social identity theory (Tajfel and Turner, 1986), and Roberts et al. (1999) revised this scale. The Revised MEIM (MEIM-R) consists of 12 items and has two sub-scales: Exploration and Commitment. The Exploration sub-scale consists of five items, such as learn more about ethnic groups, try to understand the history, and think about the meaning of ethnic background. The commitment sub-scale consists of seven items, such as attachment and belonging to an ethnic group. The MEIM-R scale has been validated in China (Gao and Sa, 2017), and we further confirmed the translation based on previous studies.

Participants were asked to rate the declarative sentences on a four-point Likert scale ranging from 1 = strongly *disagree* to 4 = *strongly agree*. Mean scores were calculated, and higher scores indicated a stronger ethnic identity. Previous studies have shown good psychometric properties in different students and groups (Roberts et al., 1999). In our study, the internal consistency of the revised scale was 0.81, indicating good reliability.

#### Self-Esteem

The first Chinese version of the Rosenberg Self-esteem Scale was published in the Mental Health Rating Scale Manual, revised by Wang et al. (1999). The scale is widely used and mainly consists of five positively descriptive questions and five negatively descriptive questions, which were reverse scored. Items are rated on a four-point Likert scale ranging from 1 = strongly *disagree* to 4 = *strongly agree*. The total score was calculated, with higher scores representing higher self-esteem.

### Statistical Analysis

The Statistical Package for Social Science (SPSS) version 22.0 was used to compute descriptive statistics, correlations, and analysis of variance (ANOVA). Structural equation modeling (SEM) was used to evaluate the hypotheses, using maximum likelihood with Analysis of Moments Structures (Amos 22.0). Model evaluation based on criteria such as CMIN/DF (χ^2^ divided by degree of freedom), where smaller values are preferable, Goodness-of-Fit Index (GFI) ≧0.9, which shows the relative amount of variance and covariance, comparative fit index (CFI) ≥ 0.9, and root mean square error of approximation (RMSEA) ≤ 0.8 has been recognized as one of the most informative criteria in covariance structure modeling.

## Results

### Descriptive Analysis

The bivariate correlations, means, and standard deviations of the primary study variables are presented in Table 2. The results indicated that cultural socialization messages were positively correlated with other variables (*r* = between 0.20 and 0.48, *p* < 0.005), and the promotion of harmony messages were positively correlated with other variables (*r* = between 0.24 and 0.49, *p* < 0.005), except for the promotion of distrust. Notably, preparation for bias was positively correlated with ethnic identity (*r* = 0.21, *p* < 0.05) and negatively correlated with self-esteem (*r* = −0.074, *p* < 0.005). Messages entailing distrust were only significantly correlated with lower self-esteem (*r* = −0.15, *p* < 0.005).

**TABLE 2:** 95% Confidence interval (CI) of bivariate correlation, mean, standard deviation of primary study variables.

	**1**	**2**	**3**	**4**	**5**	**6**
1. Cultural socialization	1					
2. Promotion of harmony	0.48**	1				
3. Preparation for bias	0.44**	0.31**	1			
4. Promotion of distrust	0.23**	0.02	0.62**	1		
5. Ethnic identity	0.36**	0.49**	0.21*	0.02	1	
6. Self-esteem	0.20**	0.24**	−0.074**	−0.15**	0.35**	1
Mean	2.02	2.88	1.78	1.41	2.65	26.87
Standard deviation	0.82	0.96	0.78	0.79	0.58	4.43

**p < 0.005, *p < 0.05.

One-way ANOVA revealed that Mulao students’ perceived promotion of distrust information varied significantly by gender; boys reported more distrust information than girls (*t* (467) = 3.72, *p* < 0.0001). One-way ANOVA results indicated significant effects of grades on the promotion of harmony information and ethnic identity. Senior students reported more harmony information than freshman and junior students [*F*(2, 466) = 6.27, *p* < 0.0001]. Senior students’ ethnic identity was higher than that of freshman students [*F*(2, 466) = 3.48, *p* < 0.05]. Meanwhile, one-way ANOVA results revealed a significant effect of parental education on ethnic identity; that was, students whose parents were educated to senior high school or above reported stronger ethnic identity than those whose parents were educated to primary school or junior high school level [*F*(2, 466) = 4.29, *p* < 0.05].

### Evaluation of Prospective Model

**FIGURE 1:**
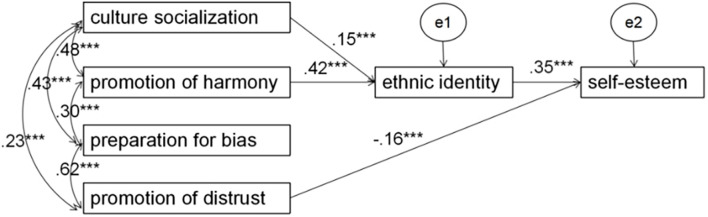
Structural equation model: χ^2^(df6) = 17.59; CMN/DF = 2.93; GFI = 0.99; CFI = 0.98; RMSEA = 0.06. ***p < 0.001. All paths are significant.

To estimate the relationships between ethnic socialization, ethnic identity, and self-esteem, confirmatory factor analysis (CFA) was conducted using AMOS 22.0, and full information maximum likelihood methods (FIML) were utilized. Because the correlation between the promotion of distrust and ethnic identity was not significant, the pathway in the model was not established. The pathway from preparation for bias to ethnic identity was deleted because the standard regression weights were not significant (*r* = 0.01). Standard solutions for SEM are presented in Figure 1. Indices of GFI were CMIN = 17.59, DF = 6, CMIN/DF = 2.93, GFI = 0.99, CFI = 0.98, and RMSEA = 0.06.

To examine the mediation effect, we used a bias-corrected bootstrapping confidence interval (95% CI) to identify significant indirect pathways. (1) The indirect effect of promotion of harmony on self-esteem through ethnic identity was significant (CI = 0.45 to 0.93), suggesting that promotion of harmony messages is associated with higher ethnic identity and subsequently related with higher self-esteem among Mulao minority students. (2) The indirect effect of cultural socialization on self-esteem through ethnic identity was significant (CI = 0.08 to 0.54), indicating that ethnic identity fully mediates the pathway from cultural socialization to self-esteem.

### Multigroup Analysis

Multigroup analysis based on AMOS 22.0 was used to evaluate whether the associations between variables were stable across gender, grades, and parental education. Comparing the Chi-square of the constrained model with structural residuals constrained to equality across groups to a baseline model with the same coefficients freely across groups, significant variance of Chi-square (Δχ^2^) indicates variation across groups (Byrne, 2009; Peng and Lu, 2020).

**FIGURE 2:**
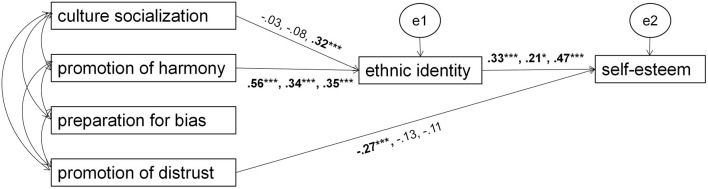
All path standardized coefficients display as the order: first grade, junior grade, senior grade. Path from cultural socialization to ethnic identity is significant only for senior grade, as the path from promotion of distrust to self-esteem is significant only for first grade students. Residual terms and their correlated errors and correlations among the four indicators of ethnic socialization are all omitted from the figure. *p < 0.5 and ***p < 0.001.

Results indicated that the variance of Chi-square (Δχ^2^) between the constrained and baseline models across gender was 25.43 (*p* = 0.09 > 0.05), and 48.61 (*p* = 0.07 > 0.05) across parental education, suggesting that the associations between variables were stable across boys and girls, and students with different parental education. However, the variance of Chi-square (Δχ^2^) between the constrained and baseline models across grades was 66.31 (*p* = 0.00 < 0.0001), indicating a significant variance in the associations across different grades. Figure 2 shows the model for three grades separately.

Further analysis used critical ratios for difference between parameters; an absolute value larger than 1.96 emphasized a significant difference between the pathway coefficients (Byrne, 2009; Peng and Lu, 2020). The results revealed that the pathway from cultural socialization to ethnic identity was significant only for senior students (B = 0.32, *P* < 0.0005). The pathway from promotion of distrust to self-esteem was significant for freshman students (B = -0.27, *P* < 0.0005).

## Discussion

We examined the relationships between ethnic socialization, ethnic identity, and self-esteem among Chinese Mulao ethnic minority junior high school students. We observed ethnic socialization in a specific dimension. The main results indicated that students who perceived more promotion of harmony messages reported stronger ethnic identity and higher self-esteem, in support of previous studies among other Chinese ethnic groups (Yin et al., 2016). Promotion of harmony is positive information, which can develop Mulao students’ positive inter-ethnic communication behaviors and relationships, further increasing their identity and sense of belonging to their ethnic group. According to social identity theory (Tajfel and Turner, 1986), self-identity is related to social groups and is a sense of belonging to the inner group that would enhance self-awareness. Therefore, ethnic identity will improve the positive evaluation of self-ability and value, thus improving their self-esteem.

Consistent with Western studies (Demo and Hughes, 1990; Hughes et al., 2006; Mohanty et al., 2006; Gartner et al., 2014; Rosnati and Ferrari, 2014; Ferrari et al., 2015), the results of the present study indicated that students who perceived more cultural socialization messages reported stronger ethnic identity, showed more positive self-concept, and higher self-esteem. Bynum et al. (2007) found that cultural pride messages predicted less psychological distress. Cultural socialization has been positively associated with self-esteem and school belonging and is inversely related to depressive and physical symptoms (Bynum et al., 2007; Rivas-Drake, 2011). In contrast to Yin et al. (2016), who found non-significant associations between cultural socialization, ethnic identity, and self-esteem among the Tibetan ethnic minority group, it may be due to the ethnic cultural difference and mainly due to different statistical methods. According to Priest et al. (2014), with awareness of the surrounding context, parents employ a range of strategies in an attempt to educate their children about their culture and prepare them for what they may encounter in their future. Cultural socialization delivers information about their group history, important people, and traditional events, which will be more likely to enhance students’ understanding of their group directly and cultivate a sense of belonging to their ethnic group.

In agreement with a previous study (Tran and Lee, 2010), the current study demonstrated that students who perceive more promotion of distrust messages were associated with lower self-esteem, which was in contrast to the findings of Gartner et al. (2014). In a longitudinal study (Gartner et al., 2014), the promotion of mistrust messages was positively related to self-esteem two years later. Considering the cultural background in our study, we found that the promotion of distrust information negatively affected students’ self-esteem. This may be due to the perceived distrust information, which hinder the harmonious interaction with other groups and affect their interpersonal relationships and self-evaluation. Guided by the risk and resilience framework, Umaña-Taylor and Updegraff (2007) also argued that various aspects of the self (i.e., self-esteem, ethnic identity, cultural orientations) can protect or enhance the risks associated with discrimination.

According to Spencer’s phenomenological variant of ecological systems theory (PVEST), young people are continually making meaning of their social worlds, including interpreting and integrating messages about their group membership and place in society, in ways that are reflected in their self-beliefs or identities (Spencer et al., 1997). Our study also verified this theory and provided deep and specific supporting evidence.

In addition, the multiple-group analysis revealed that the relationships model was stable across gender and parental education, but varied significantly across grades. The pathway from cultural socialization to ethnic identity was significant only for senior students, and that from ethnic identity to self-esteem was significant for freshman and senior students. Child correlates such as gender and age, parent correlates such as education and warmth of parent−child relationship, and situational correlates such as cultural event participation influence how often family members discuss children’s ethnic heritage with them (Brown et al., 2007), which was consistent with the results of ANOVA. Similarly, a recent study in China among other ethnic groups also showed significant age differences (Qian et al., 2015). According to Erikson (1968), self-identity is an important issue during adolescence, and individuals will continue to explore the meaning of themselves (Erikson, 1968). For ethnic individuals, ethnic identity is also a very prominent component of self-identity. Meanwhile, age is also an important factor affecting perceived ethnic socialization (Marshall, 1995). With the maturity of cognitive ability and perception of information, parents may communicate more frequently with teenagers about their ethnic group and membership. Moreover, senior students begin to think about their self-identity and look for information related to their ethnic group, which will implicitly promote ethnic identity and self-esteem.

It is important to acknowledge the limitations of our study. First, we mainly used questionnaires to derive information and self-reported data to analyze the relationships between the variables. The assessment of ethnic socialization has largely used self-report surveys, suggesting the need for the integration of other methods (Yasui, 2015). Some studies have used interviews, observation methods, and sharing stories (Juang and Syed, 2014; Reynolds and Gonzales-Backen, 2017). Adopting other sources of measurements to obtain more information will yield better results in further research. Second, the sample size was relatively small. The results of the relationships model could have been better and more reliable if the sample size was larger.

Despite these limitations, our study represents an initial effort to examine the ethnic socialization process that influences the associations between ethnic identity and self-esteem among Chinese Mulao ethnic minority students. Greater attention to ethnicity in research on youth programs can provide insight into the relevance of positive youth development for specific groups (Williams and Deutsch, 2016). Meanwhile, we can infer that different ethnic socialization messages may play different roles in identifying their ethnic membership and how they value themselves.

In conclusion, the model provided herein illustrates that the positive messages during communication in ethnic families (e.g., promotion of harmony and cultural socialization) played important roles in cultivating students’ ethnic identity and self-esteem. Meanwhile, we need to pay attention to negative messages (e.g., promotion of distrust and preparation for bias) that would hinder their mental performance. At the same time, other factors such as students’ grade or age had an impact on the associations between ethnic socialization and adolescents’ ethnic identity, as well as self-esteem. The current study mainly focused on family and parents, while according to Priest et al. (2014), schoolteachers, peers, neighborhoods, and communities were also identified as influential ethnic-racial socialization agents. The existing research also indicates that neighborhood and community characteristics influence ethnic-racial identity and parental cultural socialization practices (Coll and Marks, 2009; White et al., 2018). Moreover, considering the measurement limitations, further research that investigates ethnic socialization can adopt more measurements (e.g., in-depth interviews) and expand the research agent of ethnic socialization.

## Data Availability Statement

The original contributions presented in the study are included in the article/Supplementary Material, further inquiries can be directed to the corresponding author/s.

## Ethics Statement

The studies involving human participants were reviewed and approved by Psychology Laboratory of Guangxi Normal University, China. Written informed consent to participate in this study was provided by the participants’ legal guardian/next of kin.

## Author Contributions

LK involved in recruiting the participants and conducting the experiments. LK and SN involved in analyzing and interpreting data, and drafting the article. Both authors have read and approved the final manuscript.

## Conflict of Interest

The authors declare that the research was conducted in the absence of any commercial or financial relationships that could be construed as a potential conflict of interest.

## Publisher’s Note

All claims expressed in this article are solely those of the authors and do not necessarily represent those of their affiliated organizations, or those of the publisher, the editors and the reviewers. Any product that may be evaluated in this article, or claim that may be made by its manufacturer, is not guaranteed or endorsed by the publisher.
